# BCL11B promotes T‐cell acute lymphoblastic leukaemia cell survival via the XRCC5/C11ORF21 axis

**DOI:** 10.1002/ctm2.1580

**Published:** 2024-02-05

**Authors:** Xibao Yu, Yuchen Li, Pengyue Yang, Yan Wang, Xuan Liu, Letong Cai, Jing Lai, Yue Zhang, Xianfeng Zha, Grzegorz Krzysztof Przybylski, Ling Xu, Yangqiu Li

**Affiliations:** ^1^ The First Affiliated Hospital and Institute of Hematology, School of Medicine Jinan University Guangzhou Guangdong China; ^2^ Key Laboratory for Regenerative Medicine of Ministry of Education Jinan University Guangzhou Guangdong China; ^3^ Guangzhou Municipality Tianhe Nuoya Bio‐Engineering Co. Ltd., Guangzhou 510663 Guangdong China; ^4^ Department of Clinical Laboratory, First Affiliated Hospital Jinan University Guangzhou Guangdong China; ^5^ Institute of Human Genetics Polish Academy of Sciences Poznan Poland

Dear Editor,

1

B‐cell lymphoma/leukaemia 11B (BCL11B) is an essential transcriptional regulator of T cells and is engaged in regulating vital biological processes including T‐cell development, proliferation, differentiation and survival.^1^, ^2^ Previous studies have shown that *BCL11B* exhibits abnormally high expression levels in cases of T‐cell acute lymphoblastic leukaemia (T‐ALL) and that its deletion can significantly induce apoptosis of T‐ALL cells.^3^, ^4^ However, the molecular mechanism underlying this process remains unclear. Here, we identified X‐ray repair cross complementing protein 5 (XRCC5) as a binding protein of BCL11B and XRCC5 may regulate BCL11B expression in both transcriptional and protein level to inhibit the apoptosis of T‐ALL. In addition, we also found a new target gene of BCL11B namely Chromosome 11 Open Reading Frame 21 (C11ORF21), which also has an effect on the pathogenesis of T‐ALL. Our study indicates that the XRCC5/BCL11B/C11ORF21 signalling pathway is a potential target in the treatment of T‐ALL.

In this study, immunoprecipitation and mass spectrometry (MS) were first used to screen for binding proteins of BCL11B. In three T‐ALL cell lines, CCRF, JURKAT and MOLT4, proteins binding BCL11B were identified through immunoprecipitation followed by MS (Figure 1A). Interestingly, XRCC5 was repeatedly identified in the MS data of all the three T‐ALL cell lines, and was confirmed in the CCRF and 293T cells (Figure 1B,C). This result demonstrated that XRCC5 has an interaction with BCL11B. XRCC5 was initially recognised for its role in mending double‐stranded DNA breaks; henceforth, it was deemed to promote therapeutic resistance against cancerous cells, induced by DNA‐damaging agents.^5^, ^6^, ^7^ We then found XRCC5 binds to the X1 region of BCL11B, which contains Leucine‐zipper domain that is responsible for sequence specific DNA binding (Figure 1D), indicating XRCC5 may function as a co‐transcription factor with BCL11B to modulate the downstream gene expression. In addition, we also found that *XRCC5* was significantly up‐regulated in primary T‐ALL cells and positively correlated with *BCL11B* expression (Figure 2A–D). Moreover, both *BCL11B* and *XRCC5* expressions were inhibited in CCRF and JURKAT treated with Doxorubicin or Vincristine (Figure 2E,F), suggesting that targeting the XRCC5/BCL11B signalling pathway holds promise for the diagnosis and therapeutic intervention of T‐ALL. These results suggest that expression of *XRCC5* correlated with expression of *BCL11B* and play important roles in regulating the survival of T‐ALL cells.

**FIGURE 1:**
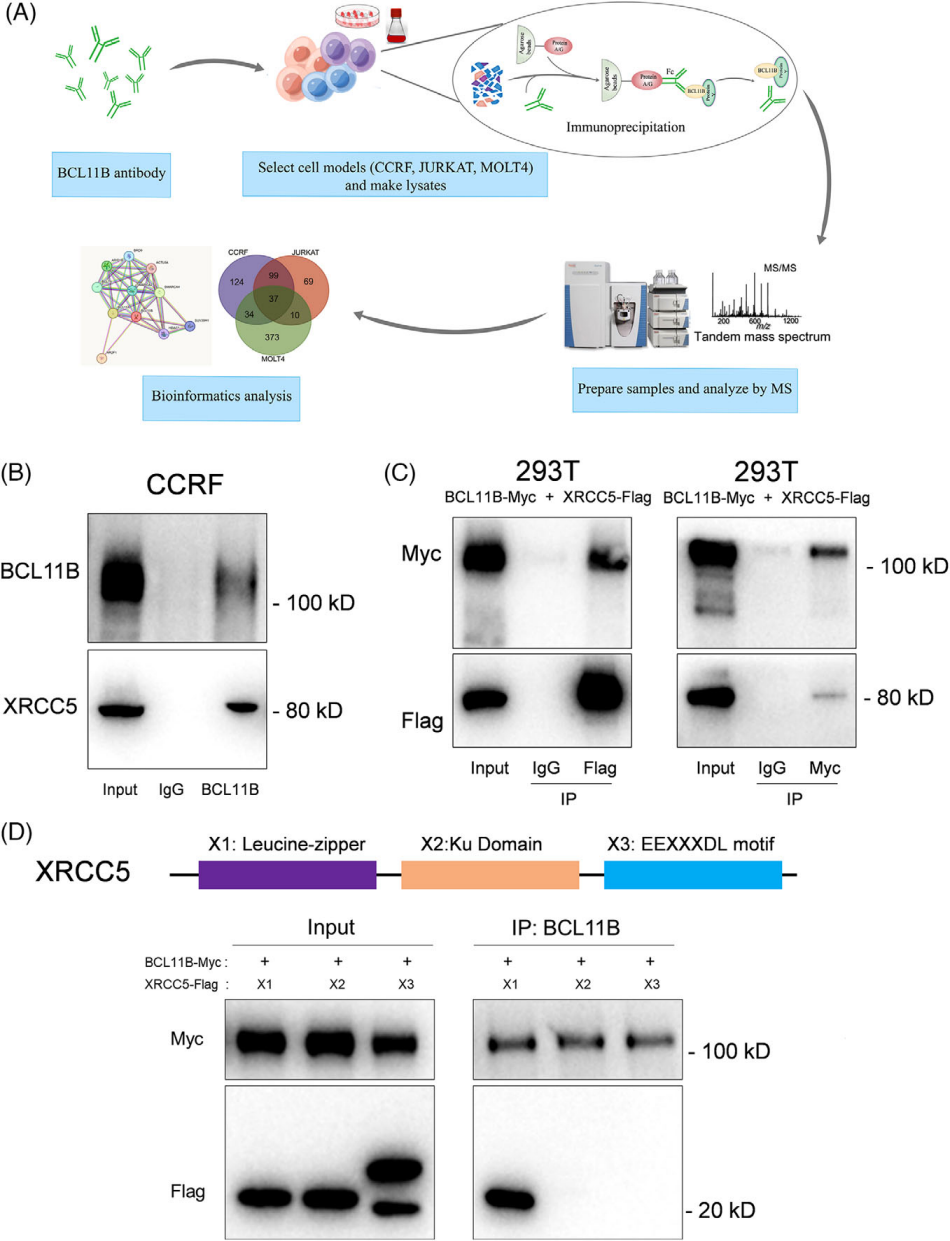
Identification of X‐ray repair cross complementing protein 5 (XRCC5) as a novel B‐cell lymphoma/leukaemia 11B (BCL11B)‐interacting protein. (A) Flow chart of immunoprecipitation (IP) tandem mass spectrometry. (B) Endogenous IP analysis of CCRF cells showed that XRCC5 is the binding protein of BCL11B. (C) Co‐immunoprecipitation (Co‐IP) analysis showed that XRCC5 was the binding protein of BCL11B; BCL11B‐Myc plasmid and XRCC5‐Flag plasmid were transfected into 293T cells, Myc beads were BCL11B protein in IP cell lysate, Flag beads was used to IP XRCC5 protein in cell lysate. (D) Co‐IP analysis showed that the X1 segment of XRCC5 can bind to BCL11B. Blots are representative of at least three independent experiments.

**FIGURE 2:**
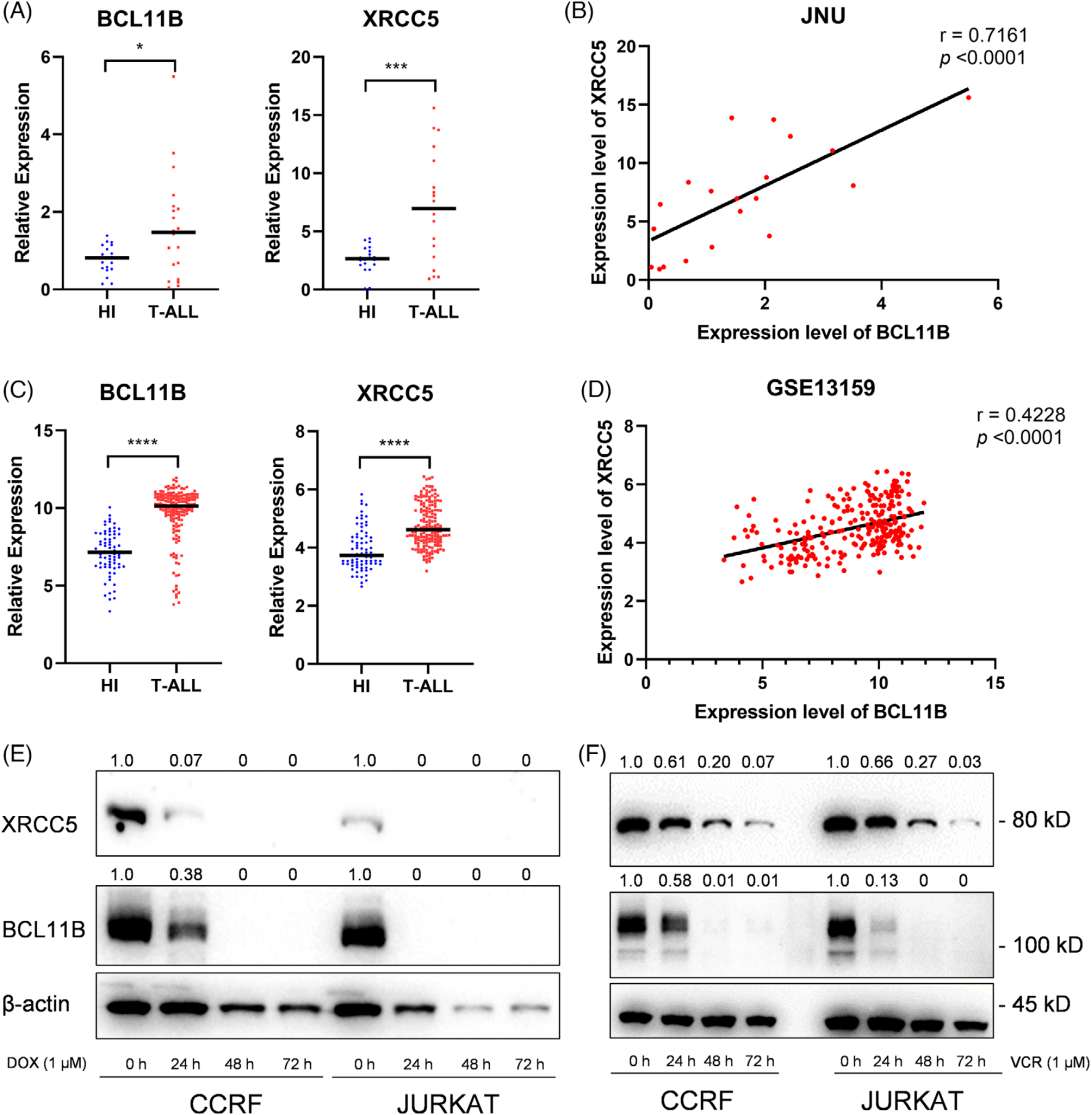
X‐ray repair cross complementing protein 5 (XRCC5) and B‐cell lymphoma/leukaemia 11B (BCL11B) are highly expressed and positively correlated in T‐cell acute lymphoblastic leukaemia (T‐ALL). (A–D) XRCC5 and BCL11B gene expression levels and correlation analysis in peripheral blood mononuclear cells (PBMC) of healthy individuals (HI) and patients with newly diagnosed T‐ALL (A and B: laboratory‐derived specimens, C and D: GSE13159 database). (E and F) The protein level of XRCC5 and BCL11B decreased with the prolongation of Doxorubicin (DOX, 1 μM) and Vincristine (VCR, 1 μM) induction time. Blots are representative of at least three independent experiments. ^*^ *p* < .05, ^**^ *p* < .01, ^***^ *p* < .001, ^****^ *p* < .0001 and ns: no significance (A and C: two‐tailed unpaired Student's *t*‐tests).

We have previously shown that inhibition of *BCL11B* expression could significantly induce apoptosis in T‐ALL cells^8^; thus, we next explored the function of *XRCC5* in the survival of T‐ALL cells. *XRCC5* was shown to be effectively knocked down by siRNA (Figure 3A,B). As expected, the knockdown of *XRCC5* promotes the apoptosis of CCRF and JURKAT cells (Figure 3C). It is noteworthy that the combination of *XRCC5* and *BCL11B* siRNA treatment significantly enhanced CCRF and JURKAT cell apoptosis compared to single‐gene siRNA (Figure 3D). In addition, down‐regulation of *XRCC5* can inhibit the expression of *BCL11B*. In contrast, inhibition of *BCL11B* has no effect on *XRCC5* (Figure S1), suggesting that *XRCC5* is an upstream regulator of *BCL11B*. Taken together, these data confirmed the involvement of *XRCC5* and *BCL11B* in T‐ALL survival.

**FIGURE 3:**
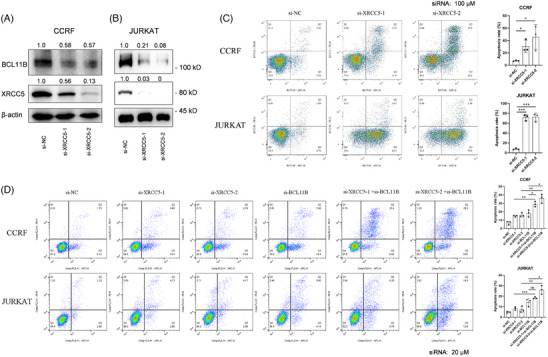
Inhibition the expression of X‐ray repair cross complementing protein 5 (XRCC5) or B‐cell lymphoma/leukaemia 11B (BCL11B) can significantly induce apoptosis of T‐cell acute lymphoblastic leukaemia (T‐ALL) cells. (A and B) Western blot detection of BCL11B and XRCC5 protein level after down‐regulation of XRCC5 expression in CCRF (A) and JURKAT (B) cells. (C) Flow cytometry analyse the apoptosis level of CCRF and JURKAT cells after down‐regulation of XRCC5 (siRNA: 100 μM). (D) Flow cytometry analyse the apoptosis levels of CCRF and JURKAT cells after XRCC5 and/or BCL11B down‐regulation (siRNA: 20 μM). A representative image of three independent experiments is shown. Values are derived from three independent experiments data are reported as mean ± standard deviation (SD). ^*^ *p* < .05, ^**^ *p* < .01, ^***^ *p* < .001, ^****^ *p* < .0001 and ns: no significance (C and D: one‐way analysis of variance [ANOVA] with Bonferroni post hoc test).

To delve deeper into the mechanism of XRCC5/BCL11B regulating T‐ALL cell survival, we took the intersection of the differential genes and the genes strongly related to *BCL11B* in the GSE13159 dataset, and differential genes in the CCRF‐siBCL11B dataset, and then *C11ORF21*, the downstream gene of BCL11B was screened (Figure 4A,B). Subsequently, we analysed the expression of *C11ORF21* in 40 samples of T‐ALL patients from our clinical centre and the data from GEO database (GSE13159). In contrast to *XRCC5* and *BCL11B*, *C11ORF21* was significantly down‐regulated in T‐ALL, and there was an inverse correlation between the expression of *C11ORF21* and that of *BCL11B* (Figure 4C–F). In addition, *C11ORF21* expression was activated when T‐ALL was treated with Doxorubicin or Vincristine (Figure 4G,H). These results suggest that BCL11B may directly inhibit the expression of *C11ORF21* which may benefit T‐ALL cell survival. Furthermore, we confirmed that down‐regulating the expression of *BCL11B* in JURKAT cells activated the expression of *C11ORF21* (Figure 4I,J). In order to determine whether BCL11B can directly transcriptionally regulate the expression of *C11ORF21*, we found multiple BCL11B binding sites in the *C11ORF21* gene through CUT‐tag combined sequencing technology (Figure 4K). Taken together, these findings suggest that *C11ORF21* serves as a novel BCL11B target gene involved in the regulation of T‐ALL cell survival.

**FIGURE 4:**
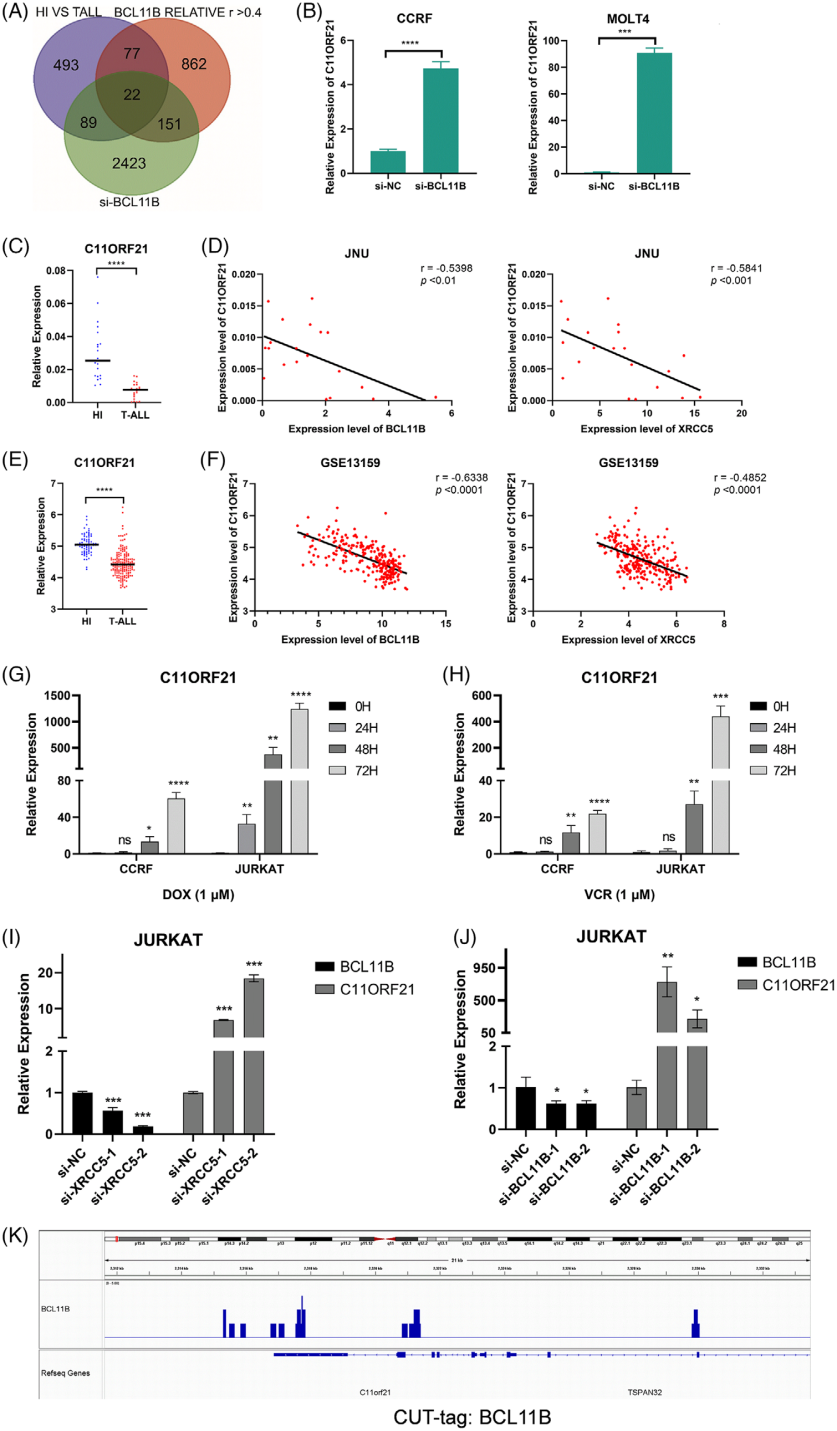
Chromosome 11 Open Reading Frame 21 (C11ORF21) is a potential target gene of B‐cell lymphoma/leukaemia 11B (BCL11B). (A) The Venn diagram shows the intersection of the differential genes between T‐cell acute lymphoblastic leukaemia (T‐ALL) versus healthy individuals in GSE13159 dataset, the genes strongly correlate with BCL11B expression (*r* > .4), and the differential genes after down‐regulation of BCL11B in CCRF cells. (B) Real‐time quantitative PCR detection of the expression of the C11ORF21 gene after down‐regulation BCL11B by siRNA in CCRF and MOLT4 cell line. (C–F) Gene expression level of C11ORF21 in peripheral blood mononuclear cells (PBMC) of healthy individuals and patients with newly diagnosed T‐ALL and the correlation analysis between C11ORF21 and BCL11B or X‐ray repair cross complementing protein 5 (XRCC5) gene in T‐ALL (C and D: laboratory samples, E and F: GSE13159 database). (G and H) The gene level of C11ORF21 increased with the induction time of Doxorubicin (DOX, 1 μM) and Vincristine (VCR, 1 μM). (I and J) Real‐time quantitative PCR detection of the gene levels of BCL11B and C11ORF21 after down‐regulating the expression of XRCC5 (I) and BCL11B (J) in JURKAT cells, the graph represents the mean and standard deviation (SD) of three independent experiments. (K) CUT‐tag combined sequencing technology analysis of the BCL11B binding site on the C11ORF21 sequence. ^*^ *p* < .05, ^**^ *p* < .01, ^***^ *p* < .001, ^****^ *p* < .0001 and ns: no significance (B, C and E: two‐tailed unpaired Student's *t*‐tests; G–J: one‐way analysis of variance [ANOVA] with Bonferroni post hoc test).

*C11ORF21* is a novel gene situated within the human chromosome 11p15.5 locus, with potential implications in Beckwith–Wiedemann syndrome and cancers.^9^ By northern blotting, this gene was found to exhibit exclusive expression solely in the human heart. Demonstration of C11ORF21–EGFP fusion protein proved that the encoding of a 132‐amino acid protein by C11ORF21, predominantly situated within the cytoplasm.^9^ Until now, the biological function of C11ORF21 has not been elucidated. Recently, Matsumoto et al. showed that C11ORF21 is a novel target gene of RUNX1, while the fusion protein RUNX1–ETO inhibited C11ORF21 expression in AML1–ETO leukaemia.^10^ These findings suggest that RUNX1 was recruited as co‐factor to around all classes of target genes of BCL11B in the T‐cell fate commitment.^1^ Thus, BCL11B and RUNX1 may bind together to repress the expression of C11ORF21 in T‐ALL, therefore, targeting C11ORF21 might be a latent way to treat T‐ALL. However, the co‐regulation of RUNX1 and BCL11B remained to be explore in the future.

Taken together, using immunoprecipitation, real‐time quantitative polymerase chain reaction, siRNA and other methods, our work suggests that XRCC5 can bind to BCL11B, subsequently suppressing C11ORF21, and facilitating the survival of T‐ALL cells (Figure S2). However, there are limitations in this study, which are needed to further investigation. First, the molecular mechanism underlying the regulation of *BCL11B* by *XRCC5* is not yet fully elucidated. Second, although XRCC5 and C11ORF21 have been found to be dysregulation in T‐ALL, their roles in vivo, along with their involvement in T‐ALL development and treatment, remain to be extensively investigated. In conclusion, the XRCC5/BCL11B/C11ORF21 axis has a crucial role in the development of T‐ALL and may serve as a promising candidate for therapeutic interventions against T‐ALL.

## AUTHOR CONTRIBUTIONS

Xibao Yu, Yuchen Li and Pengyue Yang performed the experiments, wrote the paper and analysed the data. Yan Wang, Xuan Liu and Letong Cai helped analyse the data. Jing Lai, Yue Zhang and Xianfeng Zha diagnosed and treated the patients and collected clinical samples. Grzegorz Krzysztof Przybylski helped to edit the results and revise the manuscript. Yangqiu Li, Ling Xu and Xibao Yu designed the study and wrote the manuscript. All the authors read and approved the final manuscript.

## CONFLICT OF INTEREST STATEMENT

The authors declare they have no conflicts of interest.

## ETHICS STATEMENT

The study (no. 20220223) was approved by the Ethics Committee of the Affiliated Hospitals of Jinan University.

## Supporting information

SUPPORTING INFORMATION

## Data Availability

The datasets used and/or analysed during the current study are available from the corresponding author upon reasonable request.
