# Supplementary material for: BCL11B promotes T‐cell acute lymphoblastic leukaemia cell survival via the XRCC5/C11ORF21 axis
Source: Clin Transl Med. 2024 Feb 5;14(2):e1580. doi: 10.1002/ctm2.1580 (PMC10844840; doi:10.1002/ctm2.1580)
Supplement: Supplementary file 1 — SUPPORTING INFORMATION [file CTM2-14-e1580-s001.docx]

**Supporting Information**

**BCL11B promotes T-cell acute lymphoblastic leukemia cells survival via the XRCC5/C11ORF21 axis**

Xibao Yu^1, 2#^, Pengyue Yang^1, 2#^, Yuchen Li^1, 2#^, Yan Wang^1, 2^, Xuan Liu^1, 2^, Letong Cai^1, 2^, Jing Lai^1^，Yue Zhang^1^, Xianfeng Zha^3^，Grzegorz Krzysztof Przybylski ^3^, Ling Xu^1, 2*^, Yangqiu Li^1, 2*^

^1^ The First Affiliated Hospital and Institute of Hematology, School of Medicine, Jinan University, Guangzhou, 510632, China

^2^ Key Laboratory for Regenerative Medicine of Ministry of Education, Jinan University, Guangzhou, 510632, China

^3^ Department of Clinical Laboratory, First Afﬁliated Hospital, Jinan University, Guangzhou, 510632, China

^4^ Institute of Human Genetics, Polish Academy of Sciences, Poznan, Poland

^#^These authors contributed equally to this work.

**^*^Correspondence**

Ling Xu and Yangqiu Li, Jinan University, No.601, West of Huangpu Avenue, Guangzhou 510632, Guangdong, P.R. China.

E-mail: LX: [lingxu114@163.com](mailto:lingxu114@163.com); YQL: [yangqiuli@hotmail.com](mailto:yangqiuli@hotmail.com);

**Materials and methods**

**Samples**

Peripheral blood mononuclear cells (PBMCs) from 20 patients with *de novo* T-ALL and 20 healthy individuals (HIs) were analyzed in this study (Table 1). All samples were obtained with consent, and this study was approved by the Ethics Committee of the Affiliated Hospitals of Jinan University.

**Cell culture and reagents**

The human T-ALL cell line CCRF, JURKAT, and MOLT4 cells were cultured in RPMI 1640 containing 10% fetal bovine serum. All cells were cultured in a humidified atmosphere containing 5% CO_2_ at 37°C.

Doxorubicin and vincristine were purchased from Selleck Chemicals (Shanghai, China).

**Plasmid construction**

The BCL11B and XRCC5 genes were PCR-amplified from cDNA of HIs. Then, the PCR products were purified and cloned into the PLVX eukaryotic expression vector (PLVX-BCL11B-Myc-dsRed, PLVX-XRCC5-Flag-GFP).

**Cell transfection**

The 293T cells were grown to about 90%, and subsequently digested, counted, adjusted the cell density, and then seeded into 6-well plates, according to cell culture methods. In the incubator, the 6-well plates were shaken using the "cross method" to evenly disperse the cells for overnight culture. Cell transfection was performed when cell densities reached 60%-80%. The transfection was performed using 6 μg of plasmid DNA diluted in 200 μl Opti-MEM solution, as recommended by the supplier (Yeasen Biotechnology, Shanghai, China). Add 24 μl PEI directly to the DNA dilution, pipette and mix well, and stand at room temperature for 15 min. Then gently add the transfection complex droplet dropwise to the 6-well plate. Continue to put it in a constant temperature incubator at 5% CO_2_ and 37 °C to continue to culture for 24h-48h, observe the fluorescence and collect cells and extract proteins.

**Co-immunoprecipitation (co-IP) of XRCC5 and BCL11B**

Proteins were exacted from CCRF, JURKAT and MOLT4 T-ALL cells. The proteins were incubated with a specific rabbit antibody against BCL11B (CST, USA) and a non-immune rabbit IgG (Abcam, USA) in each sample at 4°C overnight. Protein A/G agarose beads (Bimake, Shanghai, China) were used to pull down the above immune complexes. Briefly, protein A/G agarose beads were added to the immune complexes and incubated at room temperature for 3 hours. Beads were then washed with ice-cold PBS containing protease inhibitors for 3 times. Loading buffers were added to the beads and boiled for 10 min, and then centrifuged for 1 min. Supernatant containing immunoprecipitated proteins of interest was subsequently separated by SDS-PAGE and detected by Western blot.

**Western blotting**

For preparation of whole cell extracts, treated cells were washed twice in PBS and lysed on ice for 20 min in RIPA buffer with protease inhibitors. Protein extracts were separated by SDS-PAGE and then transferred to a polyvinylidene fluoride membrane. The blots were incubated with anti-BCL11B (12120S, CST), anti-XRCC5 (PTM-5405, PTM BIO), anti-Flag (14793S, CST), anti-Myc (AF0033, Beyotime) and anti-β-actin (66009-1-Ig, Proteintech) antibodies. Chemiluminescent reactions were performed using the Immobilon™ Western Chemiluminescent HRP Substrate (Millipore, USA).

**RNA interference**

CCRF and JURKAT cells were transfected utilizing the Neon® Transfection System (Invitrogen, CA, USA) with a dosage of 100 pmol oligonucleotides for the single gene knockdown or 20 pmol oligonucleotides for the double gene knockdown. Transfection was performed as previously described.[^1^](#_ENREF_1)^,^[^2^](#_ENREF_2) Small interfering RNAs (siRNAs) and negative control siRNA (siN05815122147) were purchased from RiboBio (Guangzhou, China). Sequences for siRNA are available in Table 2.

**Gene expression detection by quantitative real-time PCR (qRT-PCR)**

RNA was extracted with TRIzol reagent (Invitrogen, CA, USA), and first-strand cDNA was generated using High-Capacity cDNA Reverse Transcription Kit (Applied Biosystems, CA, USA) following the manufacturer’s instructions. qRT-PCR was performed with SYBR Green (TIANGEN, Shanghai, China) according to the manufacturer’s instructions. Gene expression levels were normalized to an *ACTB* internal control. Primers are listed in Table 3. The qRT-PCR program was as follows: 95°C for 15 min followed by 40 cycles at 95 °C for 10 s and 60 °C for 30 s.

**Annexin V and propidium iodide (PI) staining**

Cells with different treatments were harvested and washed with chilled PBS and then stained with the Annexin-V-APC/PI Apoptosis Detection Kit (MultiSciences, Shanghai, China). Analysis was performed by flow cytometry according to the manufacturer's protocol.

**CUT&Tag assay**

The CUT&Tag assay was performed by Hieff NGS® G-Type In-Situ DNA Binding Profiling Library Prep Kit for Illumina (Yeasen Biotechnology, Shanghai, China) following the manufacturer’s guidance. Briefly, 100,000 JURKAT cells were harvested and added activated paramagnetic Concanavalin A beads, followed by incubation with the primary antibody against BCL11B (12120S, CST) or secondary antibody at room temperature (RT). Cells were washed twice with magnet stand to remove unbound antibodies and incubated with 1 μl pA/G-Tn5 adapter complex for 1 h at RT. subsequently, cells were incubated in Tagmentation Buffer at 37 °C for 1 h, and then DNAs were extracted using a DNA purification kit. Libraries were prepared using the Hieff NGS Tagment Index Kit for Illumina and pooled together for paired-end 150–base pair (bp) sequencing on a NovaSeq (Novogene). Raw fastq files were trimmed using Trim Galore and aligned to the human genome (hg38) using Bowtie2. Reads were sorted and converted to BAM format, and data track visualization occurred using Integrative Genomics Viewer (IGV).

**Statistical analysis**

Data are expressed as the mean ± SD of three independent experiments. Statistical analysis was executed using GraphPad Prism 8 software. The significance of differences between groups was determined by the Student’s *t* test (unpaired and two-tailed) or one-way ANOVA with Bonferroni post hoc test for multiple comparisons. A *P* value <0.05 was considered significant. *, *P* < 0.05, **, *P* < 0.01, ***, *P* < 0.001, *P* < 0.0001, and ns, no significance.

**References**

1. Yu X, Mansouri A, Liu Z, et al. NRF2 activation induced by PML-RARalpha promotes microRNA 125b-1 expression and confers resistance to chemotherapy in acute promyelocytic leukemia*.* *Clin Transl Med*. 2021; 11: e418.

2. Yu X, Liu X, Liu X, et al. Overexpression of CASP1 triggers acute promyelocytic leukemia cell pyroptosis and differentiation*.* *European Journal of Pharmacology*. 2023; 945.

**Table 1:** Clinical information relevant to samples in Figure 2A and Figure 4C

| **ID** | **group** | **gender** | **ages** | **types** | **Used in Figure** |
| --- | --- | --- | --- | --- | --- |
| 1 | HI | F | 18 | PBMC | 2A, 4C |
| 2 | HI | M | 21 | PBMC | 2A, 4C |
| 3 | HI | F | 43 | PBMC | 2A, 4C |
| 4 | HI | M | 42 | PBMC | 2A, 4C |
| 5 | HI | M | 16 | PBMC | 2A, 4C |
| 6 | HI | F | 25 | PBMC | 2A, 4C |
| 7 | HI | F | 37 | PBMC | 2A, 4C |
| 8 | HI | F | 37 | PBMC | 2A, 4C |
| 9 | HI | M | 56 | PBMC | 2A, 4C |
| 10 | HI | M | 36 | PBMC | 2A, 4C |
| 11 | HI | M | 71 | PBMC | 2A, 4C |
| 12 | HI | F | 71 | PBMC | 2A, 4C |
| 13 | HI | M | 50 | PBMC | 2A, 4C |
| 14 | HI | M | 36 | PBMC | 2A, 4C |
| 15 | HI | M | 31 | PBMC | 2A, 4C |
| 16 | HI | M | 27 | PBMC | 2A, 4C |
| 17 | HI | F | 39 | PBMC | 2A, 4C |
| 18 | HI | F | 49 | PBMC | 2A, 4C |
| 19 | HI | M | 37 | PBMC | 2A, 4C |
| 20 | HI | F | 43 | PBMC | 2A, 4C |
| 21 | T-ALL | M | 4 | PBMC | 2A, 4C |
| 22 | T-ALL | M | 40 | PBMC | 2A, 4C |
| 23 | T-ALL | M | 22 | PBMC | 2A, 4C |
| 24 | T-ALL | F | 45 | PBMC | 2A, 4C |
| 25 | T-ALL | M | 13 | PBMC | 2A, 4C |
| 26 | T-ALL | F | 55 | PBMC | 2A, 4C |
| 27 | T-ALL | M | 25 | PBMC | 2A, 4C |
| 28 | T-ALL | M | 19 | PBMC | 2A, 4C |
| 29 | T-ALL | M | 22 | PBMC | 2A, 4C |
| 30 | T-ALL | M | 20 | PBMC | 2A, 4C |
| 31 | T-ALL | F | 21 | PBMC | 2A, 4C |
| 32 | T-ALL | M | 18 | PBMC | 2A, 4C |
| 33 | T-ALL | F | 46 | PBMC | 2A, 4C |
| 34 | T-ALL | M | 31 | PBMC | 2A, 4C |
| 35 | T-ALL | F | 21 | PBMC | 2A, 4C |
| 36 | T-ALL | M | 78 | PBMC | 2A, 4C |
| 37 | T-ALL | F | 4 | PBMC | 2A, 4C |
| 38 | T-ALL | M | 58 | PBMC | 2A, 4C |
| 39 | T-ALL | F | 5 | PBMC | 2A, 4C |
| 40 | T-ALL | F | 46 | PBMC | 2A, 4C |

**Table 2:** siRNA sequences

| Gene target | Sense (5' -> 3') | Antisense (5' -> 3') | |
| --- | --- | --- | --- |
| si-XRCC5-1 | CGUGGGCUUUACCAUGAGUAA | | UUACUCAUGGUAAAGCCCACG |
| si-XRCC5-2 | CCUCAUAUCAAGCAUAACUAU | | AUAGUUAUGCUUGAUAUGAGG |
| si-BCL11B-1 | GUCCCAAGCAGGAGAACAU | | AUGUUCUCCUGCUUGGGAC |
| si-BCL11B-2 | GCACAACAUGCAAGCAGCCCUUC | | GAAGGGCUGCUUGCAUGUUGUGC |
| si-NC | siN05815122147 | |  |

**Table 3:** Primer sequences

| Gene target | Sense (5' -> 3') | Antisense (5' -> 3') | |
| --- | --- | --- | --- |
| *ACTB* | TTGTTACAGGAAGTCCCTTGCC | | ATGCTATCACCTCCCCTGTGTG |
| *BCL11B* | TCTCACCCACGAAAGGCATCTG | | ATTTGACACTGGCCACAGGT |
| *XRCC5* | GTTCTAAAGGTCTTTGCAGCAAGA | | AAAAGCCACGCCGACTTGAGGA |
| *C11ORF21* | CTTGTCATCTCAAAGTGGCGTCG | | CAGCTGCAGGTGGCATCATTGA |

**Supplementary Figures**


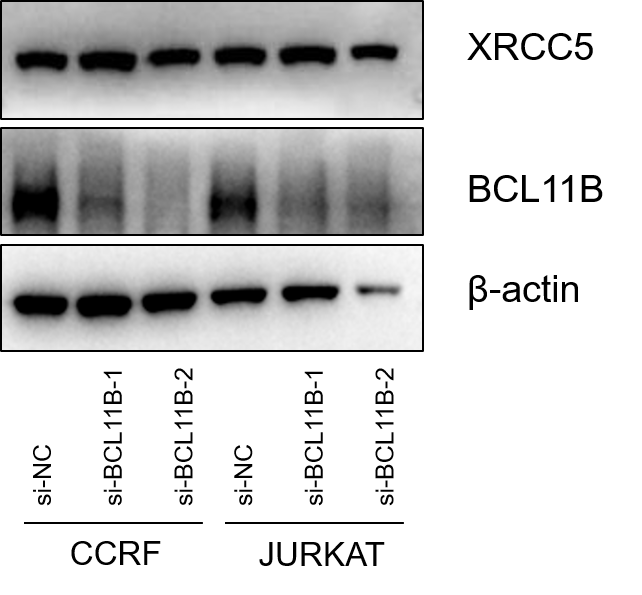


**Figure S1. Inhibition of BCL11B has no effect on XRCC5.** Western Blot detection of BCL11B and XRCC5 protein level after down-regulation of BCL11B expression in CCRF (left) and JURKAT (right) cells. Blots are representative of at least 3 independent experiments.

**
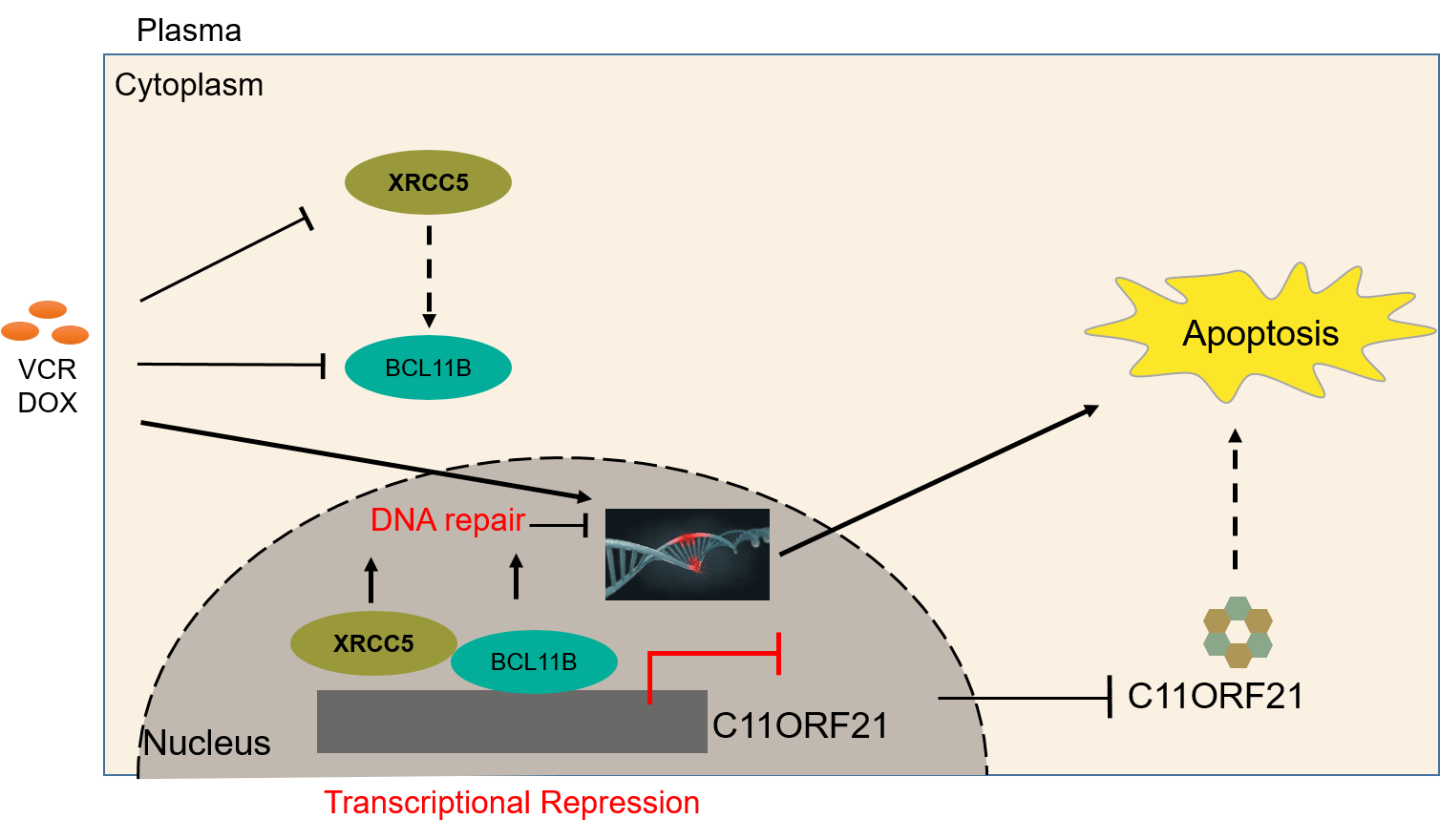
**

**Figure S2. Proposed model depicting the regulation and role of XRCC5/BCL11B/C11ORF21 axis in T-ALL.**
